# Correlates of sexual inactivity and met need for contraceptives among young women in Ghana

**DOI:** 10.1186/s12905-018-0630-0

**Published:** 2018-08-14

**Authors:** D. Yaw Atiglo, Adriana A. E. Biney

**Affiliations:** Regional Institute for Population Studies, University of Ghana, P. O. Box LG 96, Legon, Accra, Ghana

**Keywords:** Unmet need, Abstinence, Modern contraceptives, Young women, Ghana

## Abstract

**Background:**

Young women in sub-Saharan Africa continue to experience unintended pregnancies despite effective contraceptive methods being more readily available than ever. This study sought to determine the correlates of met need for contraceptives and sexual inactivity among young women in Ghana who want to postpone childbearing. We examine this among all women and then separately by marital status.

**Methods:**

Using data from 1532 females aged 15–24 years from the 2014 Ghana Demographic and Health Survey, we conducted descriptive and multinomial logistic regression analyses to assess sociodemographic, economic and obstetric determinants of the type of family planning method (current abstinence, modern contraceptive method) used by married and unmarried young women.

**Results:**

A higher proportion (~ 44%) of the respondents was currently abstinent compared to those with met need (~ 25%). Abstinence was higher among single young women while unmet and met need were higher among the married. Having at least senior high school education was significantly associated with the likelihood of current abstinence (especially among single women) and with met need. Being in the middle and rich categories, on the other hand, was associated with lower likelihood of current abstinence and a met need. Compared with multiparous women, those with one or no surviving child had a lower likelihood of being abstinent and having a met need. Other correlates of both current abstinence and met need are region of residence and ethnicity, while previous pregnancy termination and age were associated with abstinence and contraceptive use, respectively.

**Conclusions:**

Unmet need is high among young women but abstinence is an option they are using. As reproductive health programmes target the at-risk groups, the secondary and higher educational levels must be attained by most women as this is associated with use of abstinence and met need.

## Background

High risk sexual behaviour is more common among young people [1–3]; however, the negative consequences disproportionately affect females. Young women are more exposed to unintended pregnancies and the resultant unsafe abortions, severe illness, STI/HIV infections, infertility and even death as a result of unprotected sexual intercourse [4, 5]. For example, young women in Ghana have relatively higher vulnerability to the risk of unsafe abortions because they lack financial and social support for pregnancy and child care [6, 7]. In addition, pervasive challenges to the young mother and child, viz. postpartum health issues, poor birth outcomes and perinatal morbidity, are matters of concern in developing nations [8].

Unintended pregnancies prevail despite the fact that effective contraceptive methods are more readily available than ever [9]. Unmet need for modern contraceptives is persistently high among women in low and middle income countries, albeit utilisation of family planning services has continued to peak over the past few decades after the 1994 International Conference on Population and Development (ICPD) [9, 10]. Disparities exist in the uptake of services which are founded on existing socioeconomic inequalities [11]. For instance, rural residents and the poor have less access to contraceptive and safe abortion services than the urban and rich, respectively [12].

In the West African region, Ghana is a pacesetter of fertility decline, yet contraceptive uptake, even at peak levels, does not commensurate the observed declining fertility levels [10, 13]. This gap is attributed to the yet underreported abortions and traditional contraceptive use. However, an underlying deficit in such estimations of unmet need is that the measures downplay sexual inactivity or actual behaviour of women desiring to space or limit births [14], especially among younger women whose abstinence levels are high. Unmet need for family planning is pivoted on both contraceptive use and sexual activity. It is increasingly becoming evident that females in Ghana are reducing coital frequency and remaining abstinent [13, 15]. A high proportion of adolescent and young adult pregnancies globally are unintended [16], and while contraceptive use can minimise this risk of pregnancy, a sure way of minimising unmet need and pregnancy prevention is by practising abstinence [11], particularly in sub-Saharan Africa where modern contraceptive use is still low. A focus on contraceptive use among sexually active or exposed women alone obfuscates the actual behaviour of women with regards to the two main proximate determinants of young women’s fertility. Thus, understanding the correlates of preventive sexual behaviour, including contraceptive use and current abstinence, among young women is vital to enhancing the development and implementation of preventive reproductive health services [17].

While studies that investigate the correlates of contraceptive use and unmet need among sexually active young women abound, studies comparing the odds of abstinence and met need in relation to unmet need are rare. Recent studies on young women have investigated contraceptive uptake and method type, the correlates of unmet need or sexual activity among different groups of young women with various tentative results [2, 5, 18–20]. Based on findings from the aforementioned studies, we hypothesize that among young women in Ghana, there exist socio-economic, demographic, cultural and obstetric factors which differentiate access, susceptibility and exposure to sexual activity and contraception. However, none of these previous studies have determined the correlates of met need and sexual inactivity among young women, more so differentiated by marital status. We seek to address this gap in the literature with this study of young women aged 15–24 in Ghana and differentiate our analysis by marital status which is an important predictor of the profile of sexual activity and contraceptive behaviour.

## Methods

### Data

The paper drew on data from the 2014 Ghana Demographic and Health Survey (GDHS). The 2014 GDHS is the sixth and most recent nationally representative survey with in-depth information on reproductive health behaviours, socioeconomic and demographic characteristics, and child healthcare practices among men and women in their reproductive ages in line with the global DHS program [21]. The women’s questionnaire and dataset provided the necessary data on women’s background characteristics and reproductive behaviour for this study.

### Study sample

Respondents were sampled using a multilevel sampling design as indicated in the 2014 GDHS Report [21]. Ethical clearance for the survey questionnaire and data collection was provided by the Ghana Health Service Ethical Review Committee of the Ghana Health Service as well as the Institutional Review Board of ICF International, Maryland, USA. Informed consent was obtained from all participants prior to the interviews. Eligible women surveyed included those aged 15 to 49 years who resided in sampled households from 427 sampled enumeration areas across the nation. All available and willing eligible women in each of the sampled households were interviewed. Although women aged 15–24 numbered 3327, only data from a weighted sample of 1532 women who fit the criteria of being fecund, non-pregnant and had ever had sex were included in this study. The exclusion of infecund women (*n* = 33) and pregnant women (*n* = 196) from the original (unweighted) dataset was mainly on the grounds that they were not exposed to the risk of pregnancy at the time of the survey and also because retrospective recall of fertility desire may be compromised by current pregnancy status. Women also reported being postpartum amenorrheic (*n* = 254), and the majority of these were also excluded because of their relatively reduced risk of pregnancy. However, since studies indicate that the risk for pregnancy increases after 6 months postpartum [14, 22], those who reported to be amenorrheic but were more than 6 months postpartum (*n* = 84) were included in the sample.

### Variables

#### Dependent variable

The dependent variable was “contraceptive status”. Women who did not want a child within two years of the survey or were unsure about their fertility intentions but were not using a method were deemed as having an “unmet need”. Sexual activity was considered in the measure of unmet need. In their analysis of trends in contraception and sexual activity in Ghana, Machiyama and Cleland [13] found a reduction in coital frequency among women who desired to space or limit births as they were twice as likely as women who wanted a/another child soon to report being abstinent [13]. Women who had been sexually active within the past month were categorised as having an unmet need or met need based on their current contraceptive use. Following Wilson et al. [18], we defined two categories of non-users as (i) not using modern contraception but sexually active (*having an unmet need*) and (ii) not using modern contraception but sexually inactive for over one month *(currently abstinent*); and one category of users (iii) currently using modern contraception whether sexually active or not (*having a met need*).

#### Independent variables

Determinants that were significantly associated with contraceptive use in the literature were selected as covariates in this study. These were gleaned from the scoping review by Wulifan and colleagues [9]. Demographic variables included age and marital status. Socio-economic and socio-cultural variables including educational attainment, household wealth, employment status, urban-rural residence, region of residence, religion and ethnicity were also controlled for in the study. Obstetric history [2] was indicated by previous pregnancy terminations and number of living children.

### Data analyses

The data were analyzed using the statistical software package SPSS version 21. Descriptive statistics were used to assess the characteristics of the sexually active fecund but non-desiring women. Using cross tabulations, we analysed the bivariate relationships between the dependent and independent variables. We conducted chi-square tests to identify significant associations between variables. Additionally, we ran multinomial logistic regression models to determine factors associated with current abstinence and met need for contraception relative to unmet need. We further ran separate models for married and unmarried women. We maintained a 95% significance level (α=0.05) for all tests in our study.

## Results

### Descriptive statistics

As shown in Table 1, about one-third (31.0%) of respondents were not using any modern contraceptive method though sexually active. A high proportion of women were not currently sexually active (43.7%), while just over a quarter had a met need for contraception (25.3%). About a fifth of the respondents (22.9%) were married and of these about two-fifths were not using any modern method while about one-fifth were currently abstaining from sex. A little over a third of the young women (36.3%) were aged below 20 and small proportions had no education (7.8%) or only primary education (16.8%), while a third had senior high education or higher.

**Table 1:** Frequency and percent distribution of adolescent and young adult women and their contraceptive use/non-use, abstinence practice and marital status by background characteristics (Weighted sample = 1532)

Characteristic	Frequency (*n* = 1532)	Proportion of total (%)	Proportion in union (%)	Proportion with unmet need (%)	Proportion abstaining (%)	Proportion with met need / using contraceptives (%)
Unmet need for modern method						
Unmet need	475	31.0	31.1			
Abstinent	669	43.7	11.1			
Met need	387	25.3	733.2			
Marital Status				(***p*** **= .000**)		
Single	1182	77.1	0	27.7	50.3	21.9
Married	350	22.9	100.0	42.2	21.1	36.8
Age				(***p*** **= .000**)		
15–17	231	15.1	2.2	34.5	49.1	16.4
18–19	324	21.2	12.7	27.8	53.7	18.5
20–22	579	37.8	25.0	30.9	41.8	27.3
23–24	397	25.9	40.1	31.7	35.3	33.0
Educational attainment				(***p*** **= .000**)		
No education	511	7.8	59.2	38.3	30.8	30.8
Primary	258	16.8	29.5	36.4	35.7	27.9
JHS/JSS/Middle	644	42.0	24.4	32.9	42.4	24.7
Secondary/Higher	120	33.3	9.0	24.3	52.4	23.3
Wealth quintile				(***p*** **= .017**)		
Poorest	235	15.4	34.7	28.5	44.7	26.8
Poor	303	19.8	21.8	27.2	44.9	27.4
Middle	377	24.6	19.6	32.5	39.9	27.5
Rich	346	22.6	24.3	37.9	40.2	22.0
Richest	271	17.7	16.6	25.9	51.1	23.0
Type of place of residence				(*p* = .444)		
Urban	779	50.9	18.9	31.4	44.7	23.9
Rural	753	49.1	27.0	30.5	42.8	26.7
Region of residence				(***p*** **= .001**)		
Western	212	13.8	16.5	32.5	42.0	25.5
Central	163	10.6	27.0	37.7	37.0	25.3
Greater Accra	258	16.9	17.8	27.1	46.5	26.4
Volta	134	8.8	26.9	34.4	32.8	32.8
Eastern	162	10.5	24.1	36.4	45.7	17.9
Ashanti	256	16.7	18.0	28.6	50.2	21.2
Brong Ahafo	169	11.0	20.1	23.1	39.6	37.3
Northern	94	6.1	41.5	36.2	50.0	13.8
Upper East	55	3.6	34.5	25.0	48.2	26.8
Upper West	30	2.0	43.3	34.5	41.4	24.1
Religion				(*p* = .306)		
Catholic	154	10.1	26.0	26.6	47.4	26.0
Protestant / Other Christian	496	32.4	16.5	32.3	42.7	25.0
Pentecostal / Charismatic	632	41.2	23.9	29.1	43.7	27.2
Muslim	200	13.0	30.5	37.0	44.0	19.0
Other	49	3.2	32.0	34.7	38.8	26.5
Ethnicity				(*p* = .072)		
Akan	767	50.0	20.1	32.1	41.7	26.2
Ewe / Ga Dangme	343	22.4	20.4	29.4	44.0	26.5
Mole-Dagbani	238	15.5	29.8	29.8	50.4	19.7
Gursi / Gurma / Mande	118	7.7	34.5	32.8	35.3	31.9
Other	66	4.3	23.9	28.4	53.7	17.9
Employment status				(***p*** **= .027**)		
Not working	421	31.0	27.3	29.9	44.4	25.7
In school	261	17.6	2.2	28.9	52.2	18.9
Unpaid employment	233	18.6	25.9	34.0	37.9	28.1
Paid employment	616	32.7	28.1	31.5	41.8	26.7
Number of living children				(***p*** **= .000**)		
0	936	61.1	3.0	28.6	51.8	19.6
1	385	25.1	43.6	37.4	34.3	28.3
2+	211	13.8	73.5	30.3	24.6	45.0
Previous pregnancy termination				(***p*** **= .000**)		
No	1293	84.4	20.9	29.5	47.5	23.0
Yes	239	15.6	33.6	39.1	23.1	37.8
Total	1532	100.0	22.9	31.0	43.7	25.3

Computed from the 2014 Ghana Demographic and Health Survey

Bold *p*-values indicate significant associations

Approximately 18% were in the richest category of women and about a half (50.9%) resided in urban areas. The proportions of respondents residing in the ten administrative regions ranged between 2% in the Upper West and 16.9% in the Greater Accra, and the Western, Central, Ashanti, Brong Ahafo and Eastern regions each consisted of more than 10% of the sample. About 41% of women identified as Pentecostal or Charismatic, and similar to the distribution in Ghana half of the women were Akan. Regarding their employment status, about three out of every ten women were not in employment, education or training (NEET), about 18% were unemployed but still in school, 18.6% were in unpaid employment while the rest were in paid employment (32.7%). Finally, the majority (61.1%) had no living children and a much higher proportion (84.4%) had never had a pregnancy terminated.

Cross tabulations between marital status, age, educational attainment, region of residence, religion, ethnicity, employment status, number of living children and experience of pregnancy termination and the dependent variable, unmet need, were conducted. Results by marital status show that while current abstinence is higher (50.3%) among unmarried women, met need for modern contraceptives is higher among married women. Also, the proportion of young women who were abstaining roughly declines with age while the proportion with a met need increases with age. Interestingly, there was a higher proportion (53.7%) of abstinent women aged 18–19 than their younger 15–17 year old counterparts (49.1%). The latter also had the highest proportion of women with an unmet need. Across educational levels, the pattern suggests that as educational attainment increases, the proportions abstaining also increase, and they do so up till about 52.4% among women with secondary or higher education. Similarly, as educational attainment increases, the proportion with an unmet need declines. However, it was surprising to find that the largest proportion using contraception had no education, though this group had the lowest proportion of abstinent young women. The proportions with met need declined with level of educational attainment.

Region of residence revealed variations in unmet need, as the Ashanti Region had the largest percentage of women (50.2%) abstaining, the Brong Ahafo Region had the highest proportion with a met need (37.3%) and the Central Region had the highest with an unmet need (37.7%). The socio-cultural variables, religion and ethnicity, were not significantly associated with contraceptive status at the bivariate level of analysis. A higher proportion of women with no living children practised abstinence while a higher proportion of those with 2 or more children used contraception. Finally, a greater percentage of women with no previous pregnancy terminations were abstinent while higher proportions of those with pregnancy terminations either had an unmet (39.1%) or met (37.8%) need. Bivariate results were also conducted separately for married and unmarried women (not shown) and results showed some differences between them which may be due to the different rates of abstinence and met or unmet need between the two groups of women.

### Multivariate analyses

Results of multinomial logistic regression analyses shown in Table 2 indicate a strong relation between educational attainment, wealth, and number of living children for both current abstinence and use of modern methods of contraception. Associated with only abstinence are marital status, ethnicity and previous pregnancy termination while age and region of residence are only related with met need.

**Table 2:** Predicted odds from multinomial logistic regression analyses of abstinence and met need for modern contraceptives in relation to unmet need among adolescent and young adult females aged 15–24 in Ghana, by selected characteristics and according to marital status

Characteristic	All women (*n* = 1532)		Single women (*n* = 1182)		In union women (*n* = 350)	
	Abstinence	Met need	Abstinence	Met need	Abstinence	Met need
	OR (95% CI)	OR (95% CI)	OR (95% CI)	OR (95% CI)	OR (95% CI)	OR (95% CI)
Marital Status						
Single	3.342 (2.196, 5.086)***	1.437 (.954, 2.163)				
Married (r)	1.000	1.000				
Age						
15–17	0.994 (.598, 1.652)	.535 (.292, .978)*	1.026 (.582, 1.808)	.509 (.255, 1.015)	4.900 (.241, 99.680)	1.262 (.108, 14.821)
18–19	1.390 (.915, 2.109)	.723 (.451, 1.159)	1.389 (.850, 2.272)	.617 (.343, 1.110)	1.698 (.578, 4.988)	1.028 (.412, 2.564)
20–22	1.108 (.787, 1.562)	.877 (.617, 1.246)	1.192 (.777, 1.828)	.769 (.478, 1.236)	.908 (.439, 1.878)	.843 (.474, 1.498)
23–24 (r)	1.000	1.000	1.000	1.000	1.000	1.000
Educational attainment						
No education (r)	1.000	1.000	1.000	1.000	1.000	1.000
Primary	1.045 (.580, 1.883)	1.029 (.563, 1.883)	1.449 (.644, 3.262)	.625 (.253, 1.542)	.678 (.227, 2.026)	1.913 (.584, 6.267)
JHS/JSS/Middle	1.469 (.838, 2.572)	1.282 (.713, 2.304)	2.090 (.966, 4.522)	1.023 (.439, 2.387)	1.125 (.399, 3.172)	1.857 (.743, 4.638)
Secondary/Higher	2.413 (1.306, 4.458)**	2.052 (1.059, 3.976)*	3.913 (1.764, 8.678)**	1.834 (.758, 4.437)	.465 (.103, 2.093)	1.862 (.726, 4.779)
Wealth quintile						
Poorest (r)	1.000	1.000	1.000	1.000	1.000	1.000
Poor	.851 (.527, 1.374)	.899 (.528, 1.530)	.727 (.415, 1.275)	.640 (.326, 1.254)	2.205 (.696, 6.984)	2.152 (.796, 5.820)
Middle	.585 (.351, .977)*	.710 (.404, 1.247)	.451 (.248, .818)**	.589 (.293, 1.185)	2.629 (.205, 4.394)	1.412 (.467, 4.274)
Rich	.421 (.239, .742)**	.416 (.222, .782)**	.300 (.154, .583)***	.280 (.126, .621)**	1.352 (.325, 5.635)	1.049 (.330, 3.333)
Richest	.638 (.334, 1.220)	.634 (.305, 1.319)	.540 (.256, 1.139)	.641 (.264, 1.553)	2.816 (.451, 17.584)	.742 (.162, 3.403)
Type of place of residence						
Urban	.975 (.995, 1.392)	.962 (.660, 1.401)	1.111 (.760, 1.623)	.979 (.620, 1.546)	.483 (.197, 1.184)	.938 (.455, 1.936)
Rural (r)	1.000	1.000	1.000	1.000	1.000	1.000
Region of residence						
Western	.947 (.464, 1.933)	2.937 (1.194, 7.224)*	1.421 (.625, 3.232)	2.705 (.787, 9.300)	.257 (.042, 1.570)	3.379 (.753, 15.157)
Central	.796 (.372, 1.702)	2.320 (.908, 5.926)	.989 (.409, 2.391)	2.554 (.716, 9.114)	.468 (.083, 2.645)	1.503 (.322, 7.025)
Greater Accra	1.082 (.516, 2.269)	3.371 (1.352, 8.405)**	1.836 (.774, 4.358)	4.116 (1.179, 14.372)*	.161 (.026, 1.022)	1.844 (.403, 8.433)
Volta	.504 (.224, 1.133)	2.57 (.989, 6.703)	.844 (.327, 2.175)	4.092 (1.097, 15.263)*	.131 (.017, 1.018)	1.112 (.223, 5.540)
Eastern	.804 (.384, 1.684)	1.419 (.552, 3.650)	1.206 (.511, 2.845)	1.030 (.272, 3.904)	.212 (.035, 1.267)	1.673 (.383, 7.304)
Ashanti	1.462 (.718, 2.976)	2.867 (1.126, 7.296)*	2.414 (1.061, 5.494)*	3.224 (.931, 11.164)	.355 (.059, 2.131)	1.518 (.339, 6.805)
Brong Ahafo	1.221 (.591, 2.524)	5.637 (2.317,13.716)***	1.944 (.836, 4.521)	7.265 (2.154, 24.504)**	.220 (.036, 1.363)	2.498 (.590, 10.584)
Northern (r)	1.000	1.000	1.000	1.000	1.000	1.000
Upper East	.993 (.356, 2.775)	2.446 (.722, 8.292)	1.362 (.358, 5.188)	3.178 (.523, 19.297)	.358 (.061, 2.097)	1.250 (.212, 7.372)
Upper West	1.005 (.421, 2.400)	3.081 (1.088, 8.722)*	1.591 (.537, 4.716)	4.172 (.949, 18.341)	.279 (.048, 1.632)	1.570 (.319, 7.715)
Religion						
Catholic (r)	1.000	1.000	1.000	1.000	1.000	1.000
Protestant / Other Christian	.814 (.503, 1.319)	.844 (.495, 1.439)	.707 (.402, 1.235)	.673 (.342, 1.324)	.852 (.246, 2.958)	1.404 (.537, 3.665)
Pentecostal / Charismatic	1.011 (.634, 1.612)	.951 (.607, 1.701)	1.006 (.580, 1.743)	1.058 (.543, 2.060)	.942 (.299, 2.963)	1.056 (.426, 2.616)
Muslim	.643 (.365, 1.133)	.619 (.321, 1.194)	.595 (.308, 1.150)	.825 (.359, 1.895)	.367 (.129, 2.134)	.326 (.101, 1.058)
Other	.962 (.415, 2.233)	.762 (.307, 1.891)	.847 (.306, 2.332)	.780 (.236, 2.575)	2.218 (.385, 12.774)	.544 (.112, 2.643)
Ethnicity						
Akan (r)	1.000	1.000	1.000	1.000	1.000	1.000
Ewe / Ga Dangme	1.588 (1.047, 2.409)*	1.280 (.800, 2.048)	1.424 (.893, 2.272)	1.058 (.593, 1.886)	1.733 (.513, 5.854)	1.833 (.728, 4.613)
Mole-Dagbani	1.674 (1.001, 2.799)*	1.100 (.605, 1.997)	1.325 (.750, 2.340)	.678 (.331, 1.390)	3.702 (.705, 18.395)	4.436 (1.342, 14.669)*
Gursi / Gurma / Mande	.940 (.530, 1.667)	1.442 (.788, 2.637)	1.040 (.540, 2.000)	1.006 (.459, 2.204)	.272 (.040, 1.857)*	3.889 (1.219, 12.402)*
Other	2.153 (1.106, 4.190)*	1.028 (.445, 2.373)	1.963 (.924, 4.169)	.639 (.218, 1.869)	5.199 (.986, 27.419)	3.324 (.690, 16.006)
Employment status						
Not working (r)	1.000	1.000	1.000	1.000	1.000	1.000
In school	.822 (.544, 1.242)	.912 (.554, 1.500)	.884 (.570, 1.370)	.958 (.556, 1.649)	.941 (.052, 17.030)	.310 (.027, 3.582)
Unpaid employment	.739 (.505, 1.082)	.953 (.629, 1.442)	.788 (.508, 1.223)	.964 (.572, 1.626)	.768 (.308, 1.912)	1.207 (.560, 2.605)
Paid employment	1.008 (.722, 1.407)	.855 (.593, 1.233)	1.301 (.869, 1.948)	.904 (.566, 1.470)	.578 (.266,1.256)	.761 (.406, 1.425)
Number of living children						
0	.679 (.390, 1.183)	.311 (.180, .536)***	.768 (.333, 1.774)	.270 (.117, .623)**	.114 (.010, 1.287)	.583 (.194, 1.754)
1	.607 (.370, .995)*	.415 (.263, .654)***	.684 (.291, 1.610)	.403 (.173, .942)*	.718 (.352, 1.463)	.454 (.252, .818)**
2+ (r)	1.000	1.000	1.000	1.000	1.000	1.000
Previous pregnancy termination						
No (r)	1.000	1.000	1.000	1.000	1.000	1.000
Yes	.405 (.275, .596)***	1.193 (.838, 1.699)	.299 (.189, .475)***	1.115 (.704, 1.765)	.843 (.362, 1.963)	1.189 (.622, 2.270)

Computed from the 2014 Ghana Demographic and Health Survey

*OR* Odds ratio, *SE* Standard Error

*p < .05; **p < .01; ***p < .001

Compared with having an unmet need, being single is associated with an increased likelihood of being abstinent but not with meeting need for contraception. Among single women, attaining senior secondary or higher education is significantly associated with higher odds of abstinence than an unmet need. Women in the middle and rich wealth categories were less likely to be abstinent than the poorest. Further, the rich were less likely to have a met need, than an unmet need, compared to the poorest. This relationship is consistent among single women.

Respondents in the Brong Ahafo, Ashanti, Western, Upper West and Greater Accra Regions compared with those in the Northern Region, were more likely to use contraception, and this finding holds true among single women. In addition, single women in the Ashanti Region had an increased likelihood of being abstinent than having an unmet need, compared to their counterparts in the Northern Region. Ethnicity is significantly related to having a met need among married women, where the Mole-Dagbanis and Gursi/Gurma/Mandes (ethnic groups typically found in Northern Ghana) are three and two times more likely, respectively, to use contraception than not use compared to the predominant matrilineal ethnic group, the Akans. Among all women, the patrilineal Ewes and Ga/Dangmes, Mole-Dagbanis and Other ethnic groups are more likely than Akans to be abstinent compared to having an unmet need. Previous pregnancy termination is related to lower odds of being abstinent among all and single women. Finally, the likelihood of a met need also declined as number of living children declined for all and single women. However, among married women, only those with one living child had a lower likelihood of using contraception compared to having an unmet need.

## Discussion

This study details factors associated with met need for contraceptive use and abstinence among single and married young women from a nationally representative survey in Ghana. The results show that about a third of young women are at risk of unintended pregnancy. While contraceptive use is not particularly high among the sexually active there is a high prevalence of current abstinence among young women who have initiated sexual activity. Abstinence is higher among women aged 18–19 than those aged 15–17. This interesting finding indicates differences among young adults by age. A previous study about adolescent perceptions of abstinence finds marked differential perceptions by age attributed to sexual experience and cognitive development [23] and perceptions about sex are known to have some association with behaviour. Another surprising finding is that a significant proportion of women in union are currently abstinent (21.1%). This could be attributed to different factors but particularly, duolocal residential arrangements [13] which may be cultural [24, 25] or due to either partner migrating for economic or educational reasons [26]. It could also be a preferred alternative for women who are attitudinally resistant to modern contraception [13].

Education is significantly associated with abstinence and contraceptive use, as young women with senior high and secondary or higher educational levels are more likely to be abstinent as well as use modern contraceptive methods compared with women without formal education. This relationship with abstinence is significant for all young women and among single women but not when only married women are considered. These findings may indicate the educational effects of secondary or higher schooling especially on the use of abstinence to not disrupt schooling or career targets [23], particularly for those still in school, or may instil more knowledge and confidence to use contraception [2, 20]. Ghana is in the implementation phase of the Adolescent Reproductive Health (GHARH) Programme as well as the Free Senior High School Policy, thus it will be necessary to strengthen comprehensive sexual and reproductive health education among senior high and secondary level students. Household wealth significantly influences both sexual and contraception behaviour, particularly among single young women. In Ghana, there is evidence that more wealthy women may be less inclined to use modern contraception compared with their poorer counterparts [13]. In addition, we found that among young women, the wealthier are less likely to currently abstain from sex than have an unmet need.

A cultural factor related to met need is ethnicity but this relationship is significant only among married women. This may point to sociocultural attitudes surrounding fertility control within marriage [27] that makes the young married Mole-Dagbani and the Gursi/Gurma/Mande women of Northern Ghana to more likely than Akans have a met need. Incidentally, this cultural factor is also related to abstinence for all women among the major predominantly patrilineal groups (Ewes, Gas and Mole-Dagbanis) compared to the matrilineal Akan group, while among married women the Gursi/Gurma/Mande groups were the least likely to practice abstinence than have an unmet need. It would be worth further investigating sexual and reproductive behaviour among youth in relation to their culture. Closely tied in with ethnicity is the region of residence. We found that young women from the Northern Region were least likely to use modern methods while sexually active. This is to be expected as the Northern Region has the lowest rates of education and modern or any method of contraception and the highest fertility rate in Ghana [21].

Also, their obstetric history is an important predictor of young women’s current preventive sexual behaviour. The likelihood of having a met need is lower among those with one or no living child relative to multiparous women for all and single women. This finding confirms previous findings that those with no children are not necessarily encouraged to think about modern methods favourably, particularly hormonal ones [10]. They tend to use traditional or “natural” methods [10, 21], and if they do use modern methods they are most likely to use the male condom [21] which is commonly not perceived as a form of contraception [10]. Modern (hormonal) contraception is typically given to older women with children due to misconceptions about infertility resulting from use [10]. Among married women, however, the difference in contraceptive status according to number of living children only exists for women with one living child compared with multiparous women but not between the latter and those with no living children. Although the women in the study said they did not want children within the next two years, married couples would still engage in unprotected sex, especially after they had one child. This could also occur when the partner’s desire for children differs from that of the respondent. When this happens the man’s desires are generally met, as in Ghana, the marital union guarantees men rights over women to reproduction and sex [28].

Finally, ever terminating a previous pregnancy is related to lower odds of abstinence but has no significant association with met need. This contradicts the findings among young women in the US that prior gynaecologic/obstetric events including abortion could trigger behaviour change towards contraception [2] and in this case, abstinence also. The finding indicates that especially among single women, experiences with spontaneous or induced abortion are linked to having an unmet need more than being abstinent. Those with induced abortion experiences may be comfortable with their reliance on this method to terminate unintended pregnancies rather than preventing them with modern contraception [29]. Thus, they may be desensitised of the fear of an unintended pregnancy outside marriage because they can opt for an abortion. However, for those who suffered a spontaneous loss, they may be open to getting pregnant due to their previous loss, although they reported not wanting a child soon.

### Study limitations

There were a few limitations that need to be mentioned. First, we combined women using traditional contraceptive methods to those with an unmet need because the focus is on the more effective modern methods. Despite this, their traditional and folkloric methods could be effective in preventing unintended pregnancies. Another limitation is that current abstinence is measured by sexual inactivity prior to the four weeks preceding the survey. However, not having sex before the month preceding the survey does not imply that the young woman is consciously using this as her preventive method. She may have lacked the opportunity, may have suffered negative or coerced sexual debuts, or could not be sexually active for any other reason. Nonetheless, we decided that a person with sexual experience not having engaged in sexual activity for more than a month is currently abstinent, whether voluntarily or not. Finally, as expected with questions eliciting sensitive information from young people, the self-reported information may be subject to misreporting and recall biases.

## Conclusions

This study shows that sociodemographic, economic, cultural and obstetric factors are important correlates of unmet need and sexual inactivity among young women in Ghana. Young women are at risk of unintended pregnancies as approximately one-third report not wanting a child and not using modern contraception, but are sexually active. Generally, single women abstain while married women use modern contraceptives. Abstinence is not considered in most studies but can help us understand young women’s contraceptive and preventive reproductive health habits. Also, secondary and higher education is linked with use of abstinence and met need so young people must be encouraged to attain these levels. An increase in wealth also suggests more susceptibility to unintended pregnancies since wealthier women were not currently practising abstinence nor using modern contraception. Obstetric history has differing implications for sexual abstinence and met need for contraceptives by marital status. Particularly, previous pregnancy termination is linked with non-abstinence among single young women while lower number of living children is associated with less met need among all young women. One cultural factor, ethnicity, is related to abstinence among all women and contraceptive use among married women and this warrants further investigation. Targeted efforts to increase modern contraceptive uptake should improve reproductive biology education and service availability at health centres and also in schools.

## Abbreviations

DHS: Demographic and Health Surveys
GDHS: Ghana Demographic and Health Survey;
GHARH: Ghana Adolescent Reproductive Health;
ICPD: International Conference on Population and Development
NEET: Not in employment, education or training
STI/HIV: Sexually Transmitted Infection/ Human Immunodeficiency Virus
